# Living with prostate cancer: randomised controlled trial of a multimodal supportive care intervention for men with prostate cancer

**DOI:** 10.1186/1471-2407-11-317

**Published:** 2011-07-27

**Authors:** Suzanne K Chambers, Robert U Newton, Afaf Girgis, Lisa Nielsen, Stephen Lepore, Cathrine Mihalopoulos, RA Gardiner, Daniel A Galvão, Stefano Occhipinti

**Affiliations:** 1 Griffith Health Institute, Griffith University, Gold Coast, Australia; 2 Viertel Centre for Research in Cancer Control, Cancer Council Queensland, Brisbane, Australia; 3 Edith Cowan University Health and Wellness Institute, Edith Cowan University, Perth, Australia; 4 Ingham Institute, South Western Sydney Clinical School, University of NSW, Sydney, Australia; 5 Department of Public Health, Temple University, Philadelphia, USA; 6 Deakin Health Economics, Deakin University, Melbourne, Australia; 7 University of Queensland Centre for Clinical Research, University of Queensland, Brisbane, Australia; 8 Department of Urology, Royal Brisbane and Women's Hospital, Brisbane, Australia; 9 School of Psychology, Griffith University, Brisbane, Australia

## Abstract

**Background:**

Prostate cancer is the most common male cancer in developed countries and diagnosis and treatment carries with it substantial morbidity and related unmet supportive care needs. These difficulties may be amplified by physical inactivity and obesity. We propose to apply a multimodal intervention approach that targets both unmet supportive care needs and physical activity.

**Methods/design:**

A two arm randomised controlled trial will compare usual care to a multimodal supportive care intervention "Living with Prostate Cancer" that will combine self-management with tele-based group peer support. A series of previously validated and reliable self-report measures will be administered to men at four time points: baseline/recruitment (when men are approximately 3-6 months post-diagnosis) and at 3, 6, and 12 months after recruitment and intervention commencement. Social constraints, social support, self-efficacy, group cohesion and therapeutic alliance will be included as potential moderators/mediators of intervention effect. Primary outcomes are unmet supportive care needs and physical activity levels. Secondary outcomes are domain-specific and health-related quality of life (QoL); psychological distress; benefit finding; body mass index and waist circumference. Disease variables (e.g. cancer grade, stage) will be assessed through medical and cancer registry records. An economic evaluation will be conducted alongside the randomised trial.

**Discussion:**

This study will address a critical but as yet unanswered research question: to identify a population-based way to reduce unmet supportive care needs; promote regular physical activity; and improve disease-specific and health-related QoL for prostate cancer survivors. The study will also determine the cost-effectiveness of the intervention.

**Trial Registration:**

ACTRN12611000392965

## Background

Prostate cancer is the most common male cancer in developed countries (excluding non-melanoma skin cancer) [1]. In 2004 nearly 100,000 Australian men were living with a diagnosis [2]; and in Australian men aged 60 years and over, prostate cancer accounted for 4.9% of total disability-adjusted life years (DALYs) and 17.8% of cancer-related DALYs as at 2003 [3]. With increasing incidence and 10-year survival currently around 77% [2] the large cohort of men in our community living with the sequelae of diagnosis is increasing. Therefore, the well-being of men with prostate cancer is of high public health significance.

Side effects of treatment for prostate cancer include urinary incontinence, bowel and erectile dysfunction [4]. In a recent longitudinal study assessing 1649 men diagnosed with prostate cancer three years previously, relative to a healthy aged matched population, each of the main treatments led to persistent negative effects on disease-specific quality of life (QoL) [5]. Across all treatments 36% to 87% of men reported erectile dysfunction; after radical prostatectomy 12% of men had persistent urinary incontinence; and 15% of men who had external beam radiotherapy had moderate/severe bowel problems. Unmet supportive care needs are highly prevalent in men with prostate cancer: more than half (54%) of men with prostate cancer express some level of unmet psychological need and 47% express unmet sexuality needs [6]. Three of the 10 most frequently reported unmet needs relate to sexuality, three to psychological concerns, and two each in the physical/daily living and health system and information domains. Need was greatest in those with poor health; the gradients in odds ratios of unmet need were up to five-fold between best and poorest health groups. Hence there is a link between overall health and unmet supportive care needs.

Relevant to this, most men diagnosed in Australia are over 55 years of age; many experience co-morbid chronic illness such as cardiovascular disease; and many are overweight and sedentary. There is emerging evidence that obesity is related to a higher risk of biochemical recurrence and prostate-cancer specific death for men with prostate cancer [7,8]. Higher levels of physical activity and muscle strength may enhance men's ability to regain and manage urinary and bowel symptoms and improve erectile function. A cross-sectional study [9] reported that for men who received external beam radiation therapy within the past 18 months, levels of physical activity were positively associated with sexual functioning. Wolin (2009) [10] found lower incontinence in prostate cancer survivors who were normal weight and physically active compared to survivors who were obese and sedentary. They also reported that after 58 weeks post-surgery the incidence of incontinence was the same for overweight but physically active men compared to normal weight but sedentary men. Thus, interventions seeking to make a significant impact on the well-being of men who have been previously treated for prostate cancer should assess the potential for physical activity to improve outcomes.

To date no intervention research specifically targeting unmet supportive care needs after prostate cancer has been reported [11]. As well, only a few prospective exercise studies have been conducted with prostate cancer survivors [12-16], and none of these examined unmet supportive care needs. Accordingly, in this trial we apply a multimodal intervention approach that targets both unmet supportive care needs *and *physical activity. We do this by using two main strategies: self-management and group peer support. Self-management interventions have shown consistent positive effects across a range of chronic illnesses and have great potential as a cost effective method of providing support to people affected by cancer; and address issues of equity, accessibility and choice. In addition, we uniquely trial the ability of remotely-delivered, tele-based group peer support to enhance the effectiveness of self-management for men with prostate cancer. Peer support has relatively high uptake amongst men with prostate cancer with men reporting that peer discussions provide information, emotional support, and reduce feelings of social isolation [17]. We propose that adding group peer support to self-management will have increased efficacy through the mechanisms of social support; cognitive processing; lessening of stigma; and peer-based modelling of adaptive coping.

## Methods/Design

### Study Aims and Hypotheses

The overall study aim is to compare usual care to a multimodal supportive care intervention "Living with Prostate Cancer" that will combine self-management with tele-based group peer support. In doing so we will also compare the cost-effectiveness of the support intervention relative to usual care; and identify demographic, medical and psychosocial variables that predict improvement in adjustment in prostate cancer patients with the intervention approach. The study has two arms: 1) usual care; and 2) multimodal supportive care - self-management plus six months of monthly tele-based group peer support.

It is hypothesised that 3, 6 and 12 months after recruitment and commencement of the intervention:

1. Relative to men who receive usual care, men who receive the multimodal supportive care intervention will have: fewer unmet supportive care needs; greater improvements in quantity and quality of physical activity.

2. Intervention-driven improvements in unmet supportive care needs and physical activity will be mediated by self-efficacy and moderated by social constraints and social support.

3. For all men, those who show the greatest improvements over time in physical activity will also have the greatest improvements in unmet supportive needs; disease-specific and health-related QoL.

4. The multimodal supportive care intervention will be more cost-effective compared to usual care.

### Intervention

Usual care will consist of the man's standard medical management and a package containing existing evidence-based patient education materials. The multimodal supportive care intervention "Living with Prostate Cancer" will include self-management and tele-based group peer support. All men in the multimodal intervention condition will receive a printed feedback sheet with baseline assessments of unmet need, their distress thermometer score, body mass index, waist circumference and physical activity levels. This will assist them to identify potential target areas for improvement; evaluate prime concerns; and set self-management goals. As well, a password protected website will be developed utilising Web 2.0 technology to guide men through program materials and other relevant existing web-based resources in an interactive online environment. This will allow each man to access other topic areas should his needs change; locate misplaced resources; access other helpful resources; and interact with other men in the study.

The exercise module of the intervention will focus on low cost strategies that are easily implemented in any geographic area. Participants will be encouraged to meet the physical activity targets recommended for cancer survivors [18-20]. Each participant will be provided with an elastic exercise device (Gymstick, Finland) and a watch with heart rate monitor (Polar, Finland) so that all men have the basic equipment to pursue an effective exercise program even in rural or remote settings with no additional facilities.

The tele-based group peer support will be conducted through teleconference and will be co-facilitated by a nurse counsellor and experienced trained peer. A peer is a man who has been previously diagnosed with prostate cancer and who is physically and emotionally well enough to provide support to others. The focus of the peer support groups will be on sharing experiences to facilitate peer learning, coping self-efficacy, emotional/social support and processing of experiences. The groups will have up to eight members and will be formed as men are recruited. Groups will run for six months with monthly teleconferenced meetings.

### Participants

**Figure 1:**
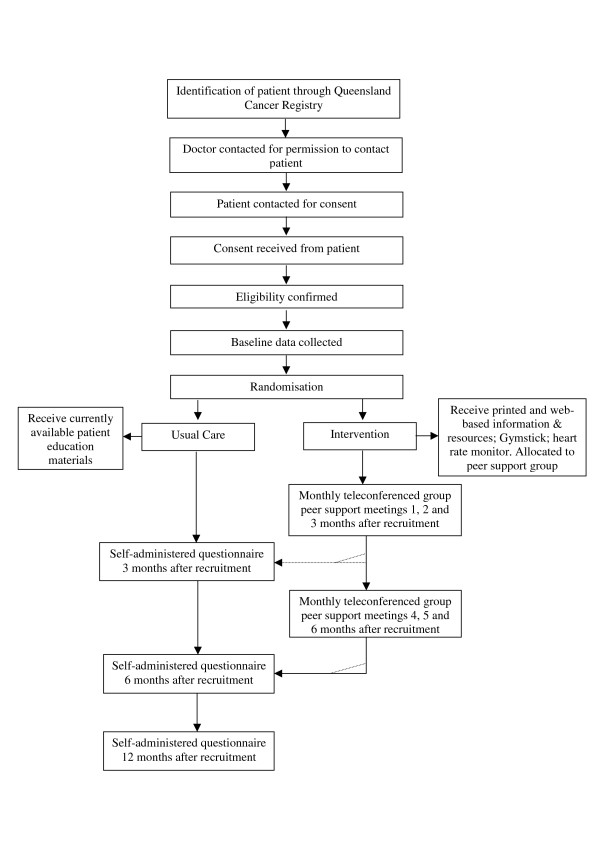
**Flowchart of recruitment, intervention and assessment**.

With the strong endorsement and support of Queensland Urologists, recruitment will be undertaken through the Queensland Cancer Registry (QCR), a population-based register of cancer diagnoses in Queensland. Clinicians will be approached for permission to contact their patient about the study. Where the doctor has given permission for contact, those patients will be contacted for consent to be in the study [21,22]. Informed written consent will be obtained before study commencement and data collection. Figure 1 illustrates the recruitment, intervention and data collection process.

Inclusion criteria are that the men must: (1) have recently been diagnosed with localised prostate cancer (men will be three to six months post-diagnosis at recruitment); (2) be able to read and speak English; (3) have no previous history of head injury, dementia or psychiatric illness; (4) have no other concurrent cancer; (5) have phone access; and (6) have physician clearance to participate in the study. The diagnosing clinician will assist in determining eligibility as part of the consent process through the QCR.

Approximately 590 men will be recruited to the study (allowing 15% attrition from treatment; 250 men in each condition will complete final assessments). According to a Monte Carlo simulation run through Mplus, this sample size will provide between 80-85% power to detect standardised intervention effect sizes of .5 (moderate) for the dichotomous outcomes even with completion rates of 70% that are lower than the projected 85%. Power for the continuous outcomes is naturally higher for the same sample sizes, as evidenced by simulations by Jo and colleagues [23].

### Study Integrity

Ethical approval has been obtained from the Griffith University Human Research Ethics Committee (Approval: PSY/F6/10/HREC). The study design will be guided by the CONSORT criteria [24]. Randomisation to study condition will occur following the completion of baseline assessment. Assessments will be by telephone and self-report written questionnaires and project staff tracking assessments will be blinded to condition where possible. Randomisation will occur in blocks of 16, with each condition randomly generated 8 times within each block to ensure an unpredictable allocation sequence with equal numbers of men in each condition at the completion of each block; and sufficient men to form a tele-based group (of 6-8) in the multimodal supportive care condition. This sequence will be undertaken by the project manager and concealed from investigators. The group sessions will be audiotaped with 25% reviewed to ensure adherence to a peer support approach. Analyses will be conducted on the basis of intention to treat.

### Measures

A series of previously validated and reliable self-report measures will be administered to men at four time points: baseline/recruitment (when men are approximately 3-6 months post-diagnosis) and at 3, 6, and 12 months after recruitment and intervention commencement. Baseline assessments will be conducted by telephone to facilitate completion of assessments prior to randomisation. Follow-up assessments will be conducted through written questionnaires that will be mailed to participants. Social constraints, social support, self-efficacy, group cohesion and therapeutic alliance will be included as potential moderators/mediators of intervention effect. Primary outcomes are unmet supportive care needs and physical activity levels. Secondary outcomes are domain-specific and health-related QoL; psychological distress; benefit finding; body mass index and waist circumference (the following list describes the measures in detail). Disease variables (e.g. cancer Gleason score, stage) will be assessed through medical and cancer registry records.

#### Moderators/Mediators

##### Coping Self Efficacy

Self-efficacy will be measured using a scale previously developed by Lepore and colleagues for men with prostate cancer [25]. This scale measures how certain men are that they will be able to control side effects, manage stress, understand their illness and communicate effectively with physicians and family.

##### Social Support

Social support will be assessed using the MOS Social Support Survey [26].

##### Social Constraints

The Social Constraints Scale (SCS) [27] will assess the extent that men perceive they cannot talk freely about cancer-related concerns and feelings with others. The SCS has good reliability and predicts adjustment to prostate cancer [28].

##### Group Cohesion

Cohesion within the peer support groups will be measured with the Perceived Cohesion Scale [29].

##### Therapeutic Alliance

The quality of the bond between the peers and men in the tele-based group peer sessions will be assessed by the Working Alliance Inventory [30].

#### Primary Outcome Variables

##### Unmet Supportive Care Needs

The Supportive Care Needs Survey Short Form 34 (SCNS-SF34) will assess patients' need for help over the last month across 5 domains: psychological, health systems and information, patient care and support, physical and daily living, and sexuality needs [31]. It has well demonstrated reliability and validity in cancer populations. An 8-item prostate cancer-specific module will be added to the standard scale [32].

##### Physical Activity

All men will complete the Godin Leisure-Time Exercise Questionnaire [33]. This questionnaire assesses the average frequency of mild, moderate and strenuous exercise during free time in a typical week. Motivational readiness for physical activity will be assessed with a four item scale that asks about current levels of physical activity and intention to become more physically active [34]. In addition, men in the intervention arm only will complete simple activity diaries assessing mode, duration and intensity of physical activity entering data from the heart rate watches provided for a seven day period at the baseline assessment before the intervention commences.

#### Secondary Outcome Variables

##### Domain Specific QoL

The International Prostate Symptom Score [35] and symptom and bother subscales of the Expanded UCLA Prostate Cancer Index [36,37] will assess disease-specific QoL.

##### Health-related QoL

The AQoL-8D examines health-related QoL over eight dimensions (e.g. independent living, mental health, relationships) and allows a simple global utility score to be calculated [38] which is essential for the derivation of Quality-adjusted-life-years (QALYs). QALYs are a useful generic outcome in economic evaluation allowing inferences regarding value-for-money to be made.

##### Psychological Distress

Brief Symptom Inventory-18 [39] will provide a global measure of current psychological distress with subscales of anxiety, depression and somatisation. Somatisation and anxiety have been found to be related to physical activity levels in colorectal cancer survivors [40].

##### Distress Screening

The single item Distress Thermometer will assess global psychological distress [41,42].

##### Positive Adjustment

Positive adjustment will be measured with the Posttraumatic Growth Inventory assessing perceived positive outcomes resulting from a diagnosis of cancer [43]. Domains include new possibilities, appreciation of life, personal strength, relating to others, and spiritual/religious change. This scale has been previously validated with cancer patients.

##### Body mass index (BMI) and waist circumference

Participants will be asked to measure their body weight and height. BMI will be calculated as weight/(height × height). Waist circumference will be measured using a cloth tape measure provided to the men with their intervention materials. Both BMI and waist circumference have been validated as indicators of overweight or obesity and correlate with cardiovascular disease and metabolic syndrome risk [44]. Self-measured height, weight and waist circumference is accurate and valid if appropriate instruction is provided [45].

##### Economic Evaluation Variables

Participants will be given a cancer care diary to fill-out with respect to all appointments and treatments they receive throughout the follow-up period. The diary includes utilisation and out-of-pocket costs for services such as hospitalisation, medication, other allied health consultations and use of alternative therapies. Items such as medications will be cross-checked against medical records to ensure all medications are captured. The diary also measures days out-of-role (including paid and unpaid work) as well as travel costs so that a broader economic societal perspective may be undertaken. These questionnaires have been found to be reliable in estimating resource use. In addition, research team and provider records will be used to determine the costs of the multimodal supportive care intervention. Measured resource use will be valued using existing unit costs from sources such as the Medical Benefit Schedule fee rates for medical attendances; AN-DRG costs of hospitalisation; and Australian Bureau of Statistics estimates of Australian earnings [46].

##### Group communication analysis

The group peer support telephone sessions will be audio-taped and transcribed to allow in-depth analysis of men's interactions during these group sessions.

### Statistical Analyses

This study is a multivariate, two-condition randomised controlled trial with repeated measures across time and continuous and dichotomous outcome variables. The analysis of longitudinal differences in outcome will use two complementary statistical approaches: multilevel (mixed) modelling (MLM) and growth mixture modelling (GMM) as applied to randomised preventative interventions by Muthén [47]. These procedures allow the testing of typical group level predictions such as Hypothesis 1 that men in the intervention condition will have better outcomes than the usual care group. However, by incorporating the hierarchical structure of assessment points nested within individual men they further permit the true assessment of individual change in unmet needs and of the individual predictors of such change (Hypotheses 2 and 3). Consequently (and unlike traditional approaches), such models deal with the heterogeneity of responses, such as that expected in the outcomes of the proposed study, by including such variation as an explicit model term. MLM and GMM have the advantages of allowing use of all available data points, which maximizes power to detect effects and reduces bias owing to missing data in longitudinal studies.

The economic evaluation will be a cost-consequences analysis conducted from both the broad societal perspective and the narrower perspective of the health care sector [48]. The economic evaluation will compare any incremental costs of the intervention (costs accrued in the intervention arm compared to costs accrued in the control arm) to the full list of incremental outcomes. The AQoL-8D will allow utility values to be determined (which are used in calculating Quality-Adjusted Life Years (QALYs)) for a cost-utility analysis, which is a more optimal design since QALYs allow comparisons across conditions. The evaluation will include only the costs of intervention delivery (excluding development or research costs), to estimate the resource use required if the multimodal supportive care intervention were rolled-out into practice. Uncertainty in the cost and outcome data and sensitivity of economic evaluation results to the methods of evaluation chosen will be tested through extensive sensitivity analyses. Depending on the results of the evaluation a longer life-time horizon may also be adopted [49].

## Discussion

This study will develop and evaluate a novel population-based intervention to reduce unmet supportive care needs; promote regular physical activity; and improve disease-specific and health-related QoL for prostate cancer survivors. To our knowledge, to date no supportive care intervention studies have targeted unmet supportive care needs for men with prostate cancer; trialled peer support as a method of enhancing self-management in this setting; or been adequately powered and designed to look differentially at individual differences [11]. This research will overcome these limitations, plus evaluate the cost-effectiveness of such an intervention. The intervention will be able to be utilised in a range of settings including broad reach tele-health support programs; health websites; and through support services and support groups internationally. This means that project outputs will be immediately translatable into practice to improve the overall well being of men with prostate cancer.

## Competing interests

The authors declare that they have no competing interests.

## Authors' contributions

SKC, RUN and AG developed the study concept and aims and initiated the project. LN, SL, CM, RAG, DAG and SO assisted in further development of the protocol. SKC was responsible for drafting the manuscript. SKC, LN and SO will implement the protocol and oversee collection of the data. All authors contributed to the final manuscript.

## Pre-publication history

The pre-publication history for this paper can be accessed here:

http://www.biomedcentral.com/1471-2407/11/317/prepub
